# Sero-prevalence and risk factors associated with African swine fever on pig farms in southwest Nigeria

**DOI:** 10.1186/s12917-015-0444-3

**Published:** 2015-06-12

**Authors:** Emmanuel Jolaoluwa Awosanya, Babasola Olugasa, Gabriel Ogundipe, Yrjo Tapio Grohn

**Affiliations:** Department of Veterinary Public Health and Preventive Medicine, Faculty of Veterinary Medicine, University of Ibadan, Ibadan, Oyo State Nigeria; Department of Population Medicine and Diagnostic Sciences, College of Veterinary Medicine, Cornell University, Ithaca, NY 14853 USA

**Keywords:** Sero-prevalence, African swine fever, Pigs, Season, Management, Transmission, Southwest Nigeria

## Abstract

**Background:**

African swine fever (ASF) is one of the major setbacks to development of the pig industry in Nigeria. It is enzootic in southwest Nigeria. We determined the sero-prevalence and factors associated with ASF among-herd seropositivity in 144 pig farms in six States from southwest Nigeria during the dry and rainy seasons using indirect Enzyme Linked Immunosorbent Assay (ELISA) for ASF IgG antibodies. An interviewer-administered questionnaire was used to collect information on demography, environmental and management factors. We performed descriptive statistics, and univariate and multivariable analyses to determine the among-herd sero-prevalence of ASF and its associated factors.

**Results:**

The overall herd sero-prevalence of ASF was 28 % (95 % Confidence interval (95 % CI) 21 – 36); it was significantly higher (P <0.05) in the dry season (54 %; 95 % CI 37 – 70) than the rainy season (18 %; 95 % CI 11 – 27). In the univariate analysis, having a quarantine/ isolation unit within 100 m radius of a regular pig pen (OR = 3.3; 95 % CI 1.3 – 8.9), external source of replacement stock (OR = 3.2; 95 % CI 1.3 – 8.3) and dry season (OR = 5.3; 95 % CI 2.2 – 12.7) were risk factors for ASF among-herd seropositivity. In the multivariable logistic regression, there was interaction between season and herd size. Our final model included season, source of replacement stock, herd size and interaction between herd size and season. Herds with an external source of replacement always had higher ASF sero-prevalence compared with herds with an internal source. The herd size effect varied between seasons.

**Conclusions:**

The ASF herd level sero-prevalence in southwest Nigeria was higher in pig herds with an external source of replacement stock and in the dry season. The effect of season of the year the samples were taken on ASF seropositivity was modified by herd size. We encourage strict compliance with biosecurity measures, especially using an internal source of replacement stock and measures that minimize movement on pig farms in southwest Nigeria, in order to enhance ASF free farms.

## Background

African swine fever (ASF) is a highly contagious and fatal viral disease of pigs caused by a DNA virus of the *Asfarviridae* family. It is a trans-boundary animal disease, defined as a disease of significant economic, trade and/or food security importance for a considerable number of countries, which can easily spread across national borders and reach epidemic proportions and for which control and management, including exclusion, require international co-operation [1].

Globally, the ASF virus is present in Africa, Italy (Sardinia), Georgia, Latvia, Poland, Ukraine, Russia (Moscow) and some Caribbean countries, with an increasing risk of spreading to ASF-free countries in Europe and America [2, 3]. African swine fever is the main threat to the pig industry in Africa because of the heavy losses incurred by pig farmers [4, 5] when it strikes, with mortality approaching 100 % [4].

Three epidemiological cycles have been recognized: the sylvatic [6], domestic [7], and sylvatic and domestic cycle [4, 8]. In Africa, all three have been reported; however, in Nigeria only the domestic cycle which maintains the ASF virus within domestic pigs is most recognized and reported [7] despite reports on detection of ASF virus in river hogs [9].

In Nigeria, the first ASF outbreak was reported in 1973 and subsequently in 1997, 1998 and 2001 [10–12]. Since the outbreak in 1997, there have been reported confirmed and unconfirmed sporadic outbreaks of ASF. African swine fever is enzootic in Nigeria [13, 14].

The pig industry in Nigeria can be classified into small holder farms – farms having fewer than 50 pigs in the herd at any point in time; medium holder farms – farms having from 50 to 100 pigs in the herd at any point in time and large holder farms – farms with over 100 pigs in the herd at any point in time. The pig farming industry in Nigeria has its largest presence in the southwest of Nigeria, with fewer high pig density areas in other geo-political zones in the country. Farming activities occur throughout the whole year with increased activities during festive periods in December. The pig production system in southwest Nigeria is predominantly confined within pig pens. The ASF scourge has however adversely affected the bustling and rising activities in this industry since the outbreak in 1997 [5]. Efforts have been made by the various State Governments through farm extension services in educating the farmers on biosecurity measures since that outbreak.

Several researchers have made efforts in contributing to the understanding of the dynamics of transmission, control and what will hopefully lead to its eventual eradication in Nigeria. Economic losses to farmers consequent to ASF scourge [5], molecular epidemiological description of the circulating strain of ASF virus in Nigeria [15, 16], surveillance of the ASF virus in both domestic and wild pig populations especially during the periods of outbreaks [9, 14, 17, 18], geographical and spatial spread of the ASF infection [19, 20] and identification of risk factors during ASF outbreaks at farm level [19] have been reported.

There had been a 5-year gap (2007–2012) between the last sero-monitoring of the ASF virus in southwest Nigeria [14]. Previous work on assessment of risk factors considered an outbreak situation; however, ASF has assumed a new enzootic status – the implication of this is that most infected farms are at subclinical level. Thus, factors responsible for the enzootic status of ASF in Nigeria are poorly elucidated.

This paper therefore attempts to address the above identified gaps of ASF herd level sero-status and unknown risk factors for ASF sero-positivity in an enzootic situation by assessing the current sero-status of pigs among herd ASF sero-prevalence and their associated factors in southwest Nigeria.

## Results

### Demography

Of 144 respondents, 108 (75 %; 95 % CI 67 – 82) were males; their mean age was 49.2 ± 14.6 years. Most of the respondents (64 %; 95 % CI 55 – 72) had tertiary education; 53 % (95 % CI 44 – 61) practiced pig farming as their only source of livelihood. The median year of practice was 7 years (Range: 1 – 36 years). Most of the farms (81 %; 95 % CI 73 – 87) were established after the last report of an ASF outbreak in 2001; the range was from 1949 to 2013. All the pig herds were raised in strict pen confinement. The median number of pigs in the herd was 45 (Range: 2 – 567). The median age of the pigs sampled was 8 months (Range: 1 – 72 months). The majority of the breeds were crosses, mainly large whites. The previous year average mortality ranged from 0 to 99 pigs: most of the pig herds (80 %; 95 % CI 72 – 86) had average mortality within 0 to 12 pigs. Only 6 (4 %; 95 % CI 2 – 9) of the pig herds had a reported history of ASF outbreak. Most of the farmers (91 %, 95 % CI 85 – 95) had access to potable water on the farm.

### ASF seropositivity

The overall herd sero-prevalence of ASF was 28 % (95 % CI 21 – 36), (40 of 144). Lagos had the lowest sero-prevalence (13 %; 95 % CI 4 – 30) while Ogun had the highest value of 57 % (95 % CI 37 – 75); there was a significant difference (*p* < 0.05) in the sero-prevalence of ASF between Lagos and Ogun States (Table 1). The sero-prevalence of ASF was higher (18 %; 95 % CI 12 – 24) in older stock (more than 12 months old) than in the younger stock (10 %; 95 % CI 7 – 13); this was significant at *p* = 0.01. The herd sero-prevalence was highest (31 %; 95 % CI 21 – 43) in small pig herds (less than 50 pigs) and lowest (21 %; 95 % CI 10 – 37) in medium herds (51 – 100 pigs); however, the difference was not significant. There was a significant difference (*p* < 0.05) in the sero-prevalence during the dry season (54 %; 95 % CI 37 – 70) and rainy season (18 %; 95 % CI 11 – 27). Overall individual crude prevalence was 78 of 657 (12 %; 95 % CI 10 – 15). Overall individual prevalence adjusted by weight of total population size was 11.2 %.

**Table 1:** Factors associated with pig herd level African swine fever seropositivity of 144 pig herds in southwest Nigeria, 2013

Variables	Seropositive n = 40 (%)	Seronegative n = 104 (%)	OR (95 % CI)^a^	P Value
State Location				
Lagos	4 (10)	27 (26)	Ref	
Ondo	4 (10)	16 (15)	1.7 (0.3; 10.3)	0.76
Ekiti	4 (10)	13 (12.5)	2.1 (0.3; 12.9)	0.58
Osun	5 (12.5)	19 (18)	1.8 (0.3; 10.1)	0.67
Oyo	6 (15)	16 (15)	2.5 (0.5; 13.9)	0.34
Ogun	17 (42.5)	13 (12.5)	8.8 (2.2; 41.8)	0.001
Total No. of pigs on farm				
Large holder farms (101 – 567)	7 (17.5)	19 (18)	Ref	
Medium holder farms (51 – 100)	8 (20)	30 (29)	0.7 (0.2; 2.8)	0.80
Small holder farms (<= 50)	25 (62.5)	55 (53)	1.2 (0.4; 3.9)	0.88
Season				
Dry	21 (52.5)	18 (17)	5.3 (2.2; 12.7)	0.00
Rainy	19 (47.5)	86 (83)		
Having slaughter slabs within 1 km radius of the farm				
Yes	5 (12.5)	23 (22)	0.5 (0.1; 1.5)	0.28
No	35 (87.5)	81 (78)		
Having rubbish heap within 1 km radius of the farm				
Yes	29 (72.5)	64 (61.5)	1.6 (0.7; 4.1)	0.30
No	11 (27.5)	40 (38.5)		
Having a quarantine/isolation unit within 100 m radius of the regular pen				
Yes	13 (32.5)	13 (12.5)	3.3 (1.3; 8.9)	0.01
No	27 (67.5)	91 (87.5)		
Farm workers having designated working clothes				
Yes	28 (70)	85 (82)	0.5 (0.2; 1.3)	0.19
No	12 (30)	19 (18)		
Taking of shower/bath at work				
Yes	26 (65)	52 (50)	1.9 (0.8; 4.3)	0.15
No	14 (35)	52 (50)		
Lending out of service boars				
Yes	8 (20)	33 (32)	0.5 (0.2; 1.4)	0.23
No	32 (80)	71 (68)		
Daily cleaning of pen floor				
Yes	36 (90)	95 (91)	0.9 (0.2; 4.0)	1.00
No	4 (10)	9 (19)		
Daily disinfection of pen floor				
Yes	8 (20)	25 (24)	0.8 (0.3; 2.1)	0.78
No	32 (80)	79 (76)		
Daily cleaning of working utensils				
Yes	28 (70)	71 (68)	1.1 (0.4; 2.6)	1.00
No	12 (30)	33 (32)		
Daily disinfection of working utensils				
Yes	9 (22.5)	21 (20)	1.1 (0.4; 3.0)	0.92
No	31 (77.5)	83 (80)		
Snacking or eating while working				
Yes	10 (25)	19 (18)	1.5 (0.6; 3.8)	0.50
No	30 (75)	85 (82)		
Wearing of work clothes outside of farm				
Yes	8 (20)	15 (14)	1.5 (0.5; 4.2)	0.56
No	32 (80)	89 (86)		
Source of replacement stock				
External source	31 (62.5)	54 (52)	3.2 (1.3; 8.3)	0.01
Internal source	9 (37.5)	50 (48)		
Feeding of swill				
Yes	22 (55)	59 (57)	0.9 (0.4; 2.1)	1.00
No	18 (45)	45 (43)		
Having carcass disposal or burying site within 1 km radius of farm				
Yes	27 (67.5)	59 (57)	1.6 (0.7; 3.7)	0.32
No	13 (32.5)	45 (43)		
Presence of nearby pig farms (within 1 km radius)				
Yes	21 (52.5)	59 (57)	0.8 (0.4; 1.9)	0.79
No	19 (47.5)	45 (43)		
Sharing of farm workers among fellow farmers				
Yes	4 (10)	13 (12.5)	0.8 (0.2; 2.8)	0.93
No	36 (90)	91 (87.5)		
Sharing of working utensils among fellow farmers				
Yes	3 (7.5)	5 (5)	1.6 (0.2; 8.7)	0.78
No	37 (92.5)	99 (95)		
Farm workers having a designated work footwear				
Yes	27 (67.5)	77 (74)	0.7 (0.3; 1.8)	0.56
No	13 (32.5)	27 (26)		

^a^Odds ratio (95 % Confidence interval)

### Herd level associated environmental and management factors to ASF seropositivity

In the univariate analysis, the presence of a quarantine or isolation unit within 100 m radius of the regular pig pen (OR = 3.3; 95 % CI 1.3 – 8.9), season of the year the samples were taken (OR = 5.3; 95 % CI 2.2 – 12.7) and source of replacement stock (OR = 3.2; 95 % CI 1.3 – 8.3) were significantly associated with ASF seropositivity (Table 1); however, the presence of pig farms within 1 km radius of another farm, having slaughter slabs or abattoir within 1 km radius of the farm, having rubbish heap or carcass disposal site within 1 km radius of the farm and presence of other animals or livestock within 100 m radius of the regular pig pen were not significantly associated with ASF seropositivity.

In the multivariable logistic regression adjusting for other covariates that were significant at *P* < 0.20 and biologically plausible ones, there was an interaction between herd size and season of the year the samples were taken. Source of restocking was a significant (OR = 2.7; 95 % CI 1.1 – 6.7) predictor for ASF herd level seropositivity. The final model included 4 predictors: season of the year the samples were taken, source of replacement stock, herd size and interaction between herd size and season of the year the samples were taken (Table 2). These were statistically significant in estimating ASF seropositivity (− 2 log-likelihood = 142.4; Goodness of fit = 0.97; *χ*2 = 20.2; *p* = 0.0005). The model correctly classified 76.5 % of the cases.

**Table 2:** Unconditional Logistic Regression of factors associated with African swine fever seropositivity of 144 pig herds with herd size as a continuous variable in southwest Nigeria, 2013

Variables	DF	β	Standard Error	Wald Chi-Square	OR^b^	95% Wald CI^c^	P-value
Intercept	1	−1.60	0.51	9.98			0.00
^a^Season (Dry/Rainy)	1	0.58	0.59	0.96			0.33
Source (External/Internal)	1	0.98	0.47	4.38	2.7	1.1; 6.7	0.04
^a^Herd size	1	−0.01	0.01	2.61			0.11
Season*Herd size	1	0.02	0.01	4.22			0.04

^a^Due to interactions between season of the year and herd size, odds ratio were not expressed because they depend on the individual value of both variables

^b^Odds ratio

^c^Confidence interval

*Interaction term

### Compliance with standard biosecurity measures

Overall average compliance with standard biosecurity measures was 61 % (95 % CI 59 – 63). Of the 144 pig herds, only 5 (3.5 %; 95 % CI 1 – 8) had functional foot dip, 113 (78.5 %; 95 % CI 71 – 85) had farm designated working clothes, 57 (40 %; 95 % CI 32 – 48) had routine pest control, 3 (2 %; 95 % CI 0 – 6) reported presence of ticks on pigs, 81 (56 %; 95 % CI 48 – 64) fed swill to their animals, 30 (21 %; 95 % CI 15 – 28) disinfected their working utensils daily, 33 (23 %; 95 % CI 16 – 31) disinfected the pen floor daily, 17 (12 %; 95 % CI 7 – 18) shared farm attendants with other pig farmers, 8 (5.5 %; 95 % CI 2 – 11) shared working utensils with other pig farmers, 41 (28.5 %; 95 % CI 21 – 37) either gave or took service boars, and 23 (16 %; 95 % CI 10 – 23) of the respondents wore their farm working clothes outside of their premises. None of these factors was significantly (*p* < 0.05) associated with ASF among herd seropositivity.

### Frequency of identified ASF related signs by respondents as occurring on the farm

The most common ASF related signs identified by the respondents were weakness or unwillingness of the pigs to stand (31 %; 95 % CI 23 – 39), followed by abortion (30 %; 95 % CI 23 – 38); the least common was reddening of ear and snout (7 %; 95 % CI 3 – 12). Reddening of ear and snout, however, was the only ASF related sign identified by the respondents that was significantly (*p* = 0.005) associated with ASF sero-positivity.

## Discussion

Our modeling indicated that source of replacement stock and season of the year the samples were taken are important determinants of ASF herd seropositivity in southwest Nigeria. However, the effect of season of the year the samples were taken on ASF herd seropositivity was modified by herd size. Herds with an external source of replacement always had higher ASF sero-prevalence compared with those with an internal source. This could be due to higher risk of introduction of an asymptomatic carrier into the herd at purchase. The spread of the ASF virus has been associated with asymptomatic carriers [21]. Introduction of ASF into a free area *via* movements of infected pigs has also been implicated in trans-boundary spread [4]. The higher ASF sero-prevalence in herds with an external source of replacement stock is also suggestive of a higher level of compromise in the bio-exclusion and biocontainment efforts, possibly due to more frequent human and vehicular movements in herds with external source of replacement stock [4].

**Fig. 1:**
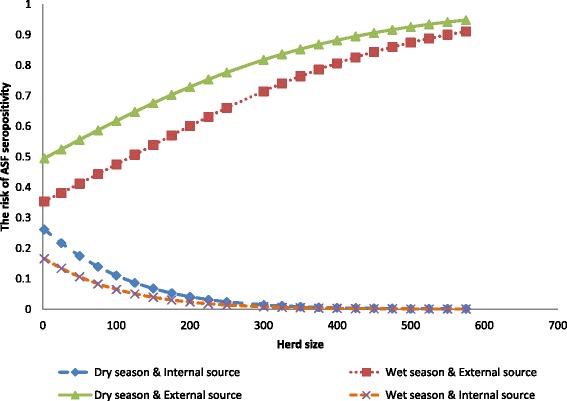
The risk of African swine fever (ASF) seropositivity in dry and wet seasons in 144 pig herds with external and internal source of replacement stock in southwest Nigeria, 2013. The risk is calculated based on the logistic regression model in Table 2. The risk of ASF seropositivity was always higher in the dry than in the rainy season and in farms with an external source of replacement stock than an internal source. The risk decreased faster in the dry season with increasing herd size in farms with an internal source of replacement stock. The risk also increased faster in the rainy season with increasing herd size in farms with an external source of replacement stock

The logistic regression technique allowed us to calculate the risk of ASF seropositivity as a function of the determinants in the final model using the formula: Prevalence (seropositive) = 1/1 + e^-[α + Season*β1 + Source*β2 + Herdsize*β3 + Season*Herdsize*β4].^ To demonstrate the interaction effect of season of the year the samples were taken and herd size we calculated the risk of ASF seropositivity for dry season and internal source of replacement, dry season and external source of replacement, rainy season and external source of replacement, and rainy season and internal source of replacement (Fig. 1). The risk of ASF seropositivity was always higher in farms with an external source of replacement stock than an internal source. Among herds with an external source of replacement the risk of ASF seropositivity was higher in the dry season than in the rainy season. Among herds with an internal source of replacement the risk of ASF seropositivity was higher in small and medium farms during the dry season than in the rainy; there was no difference in large herds. The risk decreased faster in the dry season with increasing herd size in farms with an internal source of replacement stock. The risk also increased faster in the rainy season with increasing herd size in farms with an external source of replacement stock (*i.e.*, the herd size effect was not constant between seasons). These ASF risk dynamics in pig herds in southwest Nigeria bring a new dimension to the understanding of the ASF epidemiological cycle and its enzootic status in the region. A second model considered herd size as a categorical variable; here there was no interaction between herd size and season. The likelihood of having an ASF seropositive pig herd increased by five and three times during the dry season and for farms with an external source of replacement stock respectively.

In our model, preponderance of susceptible pigs arising from increased restocking in the rainy season, especially in large herds, could explain the faster change in the risk of ASF seropositivity in the rainy season with increasing herd size in farms with an external source of replacement. The rapid reduction in ASF risk in the dry season with increasing herd size in farms with an internal source of replacement could also be explained from the above standpoint because most pig farmers dispose of their finisher stock in the dry season [22, 23]. Herd size has been associated with pig diseases [24, 25]. The higher ASF sero-prevalence in the dry season could be due to the presence of other factors that favor the prevalence and maintenance of ASF aside from restocking. The significant association between season of the year the samples were taken and source of replacement stock with ASF herd seropositivity in this study is similar to the findings of Atuhaire *et al.* [22] in a 12-year epidemiological overview of ASF in Uganda.

Numbers of movements of pigs and pig products increase in the dry season, resulting from increased trading demands during the festive period in December. Moreover, farmers have a higher propensity to dispose of mature pigs during this period because of feed scarcity and meeting of domestic (family) needs which is always on the increase during the dry season [22, 23]. Local and international trading in pigs and pig products has been associated with ASF outbreaks [2, 4, 22, 26].

Magali [27] reported insignificant but higher prevalence of ASFV in ticks during the dry than rainy season in South Africa. Although only 2 % of our respondents reported seeing ticks and their presence was not significantly associated with seropositivity in this study, this might have been under reported because of observational bias by the farmers. Thus, the role of ticks in the epidemiology of ASF in Nigeria may require further studies. Ticks play a major role in the sylvatic cycle of ASF transmission in East and Southern Africa [6], and in transmission between the sylvatic cycle and domestic pigs [4].

Another possibility could be that after infection consequent to introduction of an asymptomatic carrier by purchase in the rainy season in pig farms with an external source of replacement, the disease takes a chronic or subacute course and thus the higher sero-prevalence in the dry season. African swine fever viral infection has been reported to persist for a long time in the blood [28]. These disease dynamics may support the enzootic status of ASF in pig herds in southwest Nigeria.

In the univariate analysis, the presence of a quarantine or isolation unit within 100 m radius of the regular pig pen was significantly positively associated with ASF seropositivity. This underscores the importance of proper location of the quarantine or isolation units in order to achieve their intended purpose of biosecurity. The proximity of such units to other operational units like farrowing, fattening *etc.* could actually be a risk for having ASF seropositive farms. It is recommended that siting of the quarantine/isolation unit should not be less than 100 m from the regular pig pen [29], which is a challenge to most small holder farms in southwest Nigeria because of inadequate space due to land tenure system and financial constraints. However, when we tested the association between having quarantine/isolation unit within 100 m of the regular pig pen and ASF herd seropositivity controlling for other covariates the association was no longer significant. The effect poor siting of quarantine or isolation unit could have on herd seropositivity is, however, noteworthy.

In our study, we did not find a significant effect of environmental factors such as presence of pig farms within 1 km radius of another farm, having slaughter slabs or abattoir within 1 km radius of the farm, having rubbish heap or carcass disposal site within 1 km radius of the farm and presence of other animals or livestock within 100 m radius of the regular pig pen on ASF seropositivity. However, Fasina *et al.* [30] showed some of these factors like presence of an abattoir in a pig farming community and presence of an infected pig farm in the neighborhood to be significantly associated with ASF outbreaks. This difference could be due to the effects of environmental factors on ASF virus (ASFV) maintenance becoming insidious as the occurrence of ASF became enzootic.

We reported an overall herd ASF sero-prevalence of 28 %; this is significantly lower than the value (93 %) reported 5 years ago by Olugasa [14] across the same geographical region. The significant difference in our study sero-prevalence estimate of 28 % and the previous sero-prevalence of 93 % by Olugasa [14] which was used to determine our sample size would have slightly widened our set margin of error; however, our study sero-prevalence estimate is still within a 7 % margin of error of the population true sero-prevalence. All the other States except Ogun had a marked decline in their herd ASF sero-prevalence when compared with a 5-year value reported by Olugasa [14]. This indicates some improvement in the control measures by the Governments at all levels to eradicate the disease. The ASF herd sero-prevalence in Ogun is highest and almost stable, followed by Oyo: the high sero-prevalence in these States since the outbreak in 1997 could be because both States had international borders. Ogun was the first State in Nigeria to experience the outbreak of ASF in 1997 which spread through trans-border trade from the Republic of Benin [31]. Movement of pigs and pig products across borders from infected areas has been reported to be positively correlated with ASF seropositivity and outbreaks [4, 32]. The herd sero-prevalence is higher in small pig herds than in larger pig herds, though not significantly so; this may be due to difficulty in adhering to strict biosecurity by small holder farms or possibly less attention to simple biosecurity measures than on big farms. Moreover, the sero-prevalence of ASF is significantly higher in older stock than younger stock; this could be because restriction of movement to sections of the herds containing young pigs is greater than for older ones. It could also be that older stock had a longer time to develop antibodies to the ASF virus than younger stock, or possibly due to long persistence of ASF antibodies for a period of time after exposure [28]. There could also exist differences in ASFV transmission rates among the various age groups. Olugasa [14] also reported differences in sero-prevalence of ASF among various age groups.

Biosecurity is defined as the implementation of measures that reduce the risk of the introduction and spread of disease agents; it requires the adoption of a set of attitudes and behaviors by people to reduce risk in all activities involving domestic, captive/exotic and wild animals and their products [33]. There was no significant difference in the level of compliance with some of the biosecurity measures between seropositive and seronegative herds in our study population; however, Fasina *et al.* [30] reported significant associations between food and water control, separation/isolation of sick pigs, washing and disinfection of farm equipment and tools, consultations or visits by veterinarians/paraveterinarians, pest/rodent control, and sharing farm tools and equipment and ASF outbreaks in Nigerian farms.

With the understanding that ASF is enzootic in Nigeria, one would expect to see less apparent signs of peracute or acute forms of ASF, but rather, more of the subacute, chronic or subclinical forms. Farmers in this study reported noticing weakness or unwillingness of the pigs to stand (31 %), followed by abortion (30 %); the least common sign was reddening of the ear and snout (7 %). Reddening of the ear and snout, however, was the only ASF related sign identified by the respondents that was significantly (*p* = 0.005) associated with ASF seropositivity. There is no sign, however, that is pathognomonic to ASF. The implication of this is that the ability of the farmers to recognize such associated signs could assist early detection of an infection. However, the challenge with early detection is the non-willingness to report by the farmers. Farmers may not report because the adjudged compensation by the Government, if any, is non-commensurate. Early detection and reporting is critical to ASF control and eradication [34].

Pig farmers in Nigeria are mostly males, in their mid-50s (mean 49.2 ± 14.6 years) and most had tertiary education. More than half of the population of pig farmers studied had pig farming as their main source of livelihood. This indicates the importance of the farming sector and the socioeconomic impact adverse effects of disease such as ASF could have on livelihood. The pig sector appears to be recovering after the devastation caused by the ASF outbreak in 1997, which sent the majority of farmers out of business. Most of the farmers raised their pigs in strict pen confinement; greater risk of ASF seropositivity has been linked with free range pigs [32, 35, 36].

This study may be limited by information bias which is common to questionnaire administration, as respondents may give favoring or biased responses and not their actual practice. We mitigated this by triangulating – we designed the questionnaire in such a way that certain questions were deliberately repeated in different ways. We also envisaged interviewer bias and we reduced this by the training of the interviewers used in this study. These limitations were taken into consideration in the interpretation of the data.

## Conclusions

Our findings indicate that ASF herd level sero-prevalence in southwest Nigeria was higher in pig herds with an external source of replacement stock than an internal source; and in the dry season than in the rainy season. The effect of season of the year the samples were taken on ASF seropositivity was modified by herd size. The observed increase in ASF risk in the rainy season with increasing herd size in pig herds with an external source of replacement and observed decrease in ASF risk in the dry season with increasing herd size in pig herds with an internal source of replacement suggest that large herds are at greater risk of ASF infection *via* introduction of an asymptomatic pig than through other indirect means such as fomites, movement of vehicles and personnel. We recommend strict compliance with biosecurity measures, especially using an internal source of replacement stock and measures that minimize movement on pig farms. The associations between ASF herd level sero-prevalence and source of replacement stock and the effect of herd size on season of the year the samples were taken have implications for the understanding of ASF transmission and application in the disease modeling and in development of a suitable control and eradication strategy for ASF in Nigeria. The role of ticks either on pigs or in pens in the enzootic status of ASF in Nigeria will be an area for further investigation.

## Methods

### Study locations

**Fig. 2:**
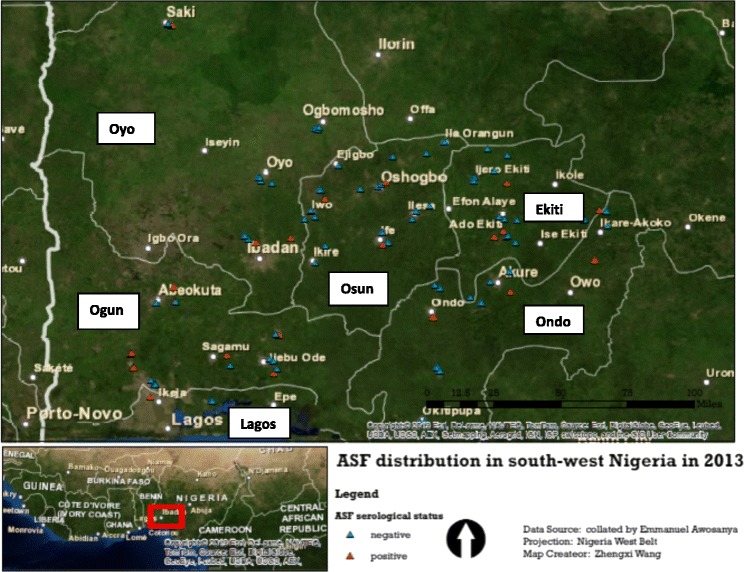
Geographical spread of 144 pig herds surveyed for African swine fever (ASF) seropositivity in southwest Nigeria in 2013. Random distribution of the pig herds is shown

The study area included six States – Lagos, Ogun, Oyo, Osun, Ondo and Ekiti (Fig. 2). The choice of the States was informed by the large presence of pig farming activities in these States and having reported outbreaks of ASF and the present enzootic status of those States. Each of the States has three senatorial districts and varying local government areas (LGAs). Pig farms from these senatorial districts and LGAs were included.

The study areas have varying sizes of pig populations: Lagos (approximately half a million pigs), Ogun (approximately half a million pigs), Oyo (approximately three hundred thousand pigs), Osun (approximately two hundred thousand pigs), Ondo (approximately a hundred thousand pigs) and Ekiti (approximately a hundred thousand pigs) [37].

Of the studied States only Ogun and Oyo have international borders with Benin Republic while Lagos is a coastal city. The location of Nigeria in Africa is shown in the inset (bottom left) in Fig. 2.

### Study design, sample size and sampling

We conducted a cross sectional study on pig farms across the six States in southwest Nigeria from November 2012 to August 2013.

A total sample size of 657 pigs from 144 pig farms were used for the study. We approached this by using the formula below [38]:

$$ \mathrm{n}=\frac{{\mathrm{Z}}^2\mathrm{p}\left(1-\mathrm{p}\right)\ast \mathrm{D}}{{\mathrm{E}}^2} $$

Where Z is the reliability coefficient put at 1.96 at a 95 % confidence interval and E is the margin of error at 5 %. Based on previous studies we powered the study to detect among herd sero-prevalence (p) of 92.9 % [14]. This gave a minimum sample size of 101 farms. We adjusted for clustering by using the formula *D* = 1 + (*b*-1) *ρ* [39], where D is the design effect, *b* is the average number of samples per cluster and *ρ* is the intra-cluster correlation coefficient. We decided to sample an average of four pigs per farm (*b* = 4), about 10 % of the pig population on the farm*.* An intra-cluster correlation coefficient of *ρ* = 0.13 was assumed based on an earlier study [40]. This gave us a *D* of 1.4. We multiplied the cluster design effect (1.4) by the earlier calculated sample size of 101 farms to arrive at a total of 141 farms. In all, a total of 144 farms and 657 pigs were sampled.

We used simple random sampling to select pig farms from a list of registered pig farms in Lagos (150) and Ogun States (124). However, for the remaining four States without a register, we used simple random sampling to select between two and four local government areas from the existing three senatorial districts in the States. At least one pig farm was chosen from each selected local government area and a total of at least six pig farms were chosen from each of the three senatorial districts. Randomness was verified using geospatial analysis by determining spatial autocorrelation (Global Moran’s I) in ArcMap version 10.2. A minimum of 20 pig farms were selected from each State except Lagos and Ogun (30 pig farms) where there is a larger presence of piggery activities and Ekiti (17 pig farms) where we had fewer pig farms. The pigs were selected using stratified sampling (piglets (weaners and growers less than or equal to 12 months old) and adult pigs (sow and boars more than 12 months old)). Equal allocation was adopted. An average of four pigs per farm was sampled.

### Data collection

#### Blood collection and Laboratory analysis

We obtained venous blood (3 – 5mls) samples from the cranial vena-cava of selected pigs. They were collected into plain tubes and transported in ice packs to the laboratory. Sera were properly labeled and stored at −20 °C until used in batches. We conducted the Indirect Enzyme Linked Immunosorbent Assay – ELISA – [41] test using the ASF kit (SVANOVIR® Sweden) to screen the pigs’ sera for ASF IgG antibodies at the Immunology laboratory, Veterinary Public Health and Preventive Medicine, University of Ibadan, Nigeria. The test kit had sensitivity and specificity of 100 % and 92.5 % respectively and can detect antibodies from day seven post infection. The samples were collected from different farms during the dry (December to March) and rainy (June and July) seasons [42] in order to detect seasonal variation. Herd sero-prevalence was determined. We defined herds as seropositive if at least one pig was seropositive in the ELISA test. We also determined the overall individual crude prevalence and adjusted it by the weight of the total population size. This was achieved by dividing the individual herd size by the total population size and multiplying by the proportion that were positive in the samples taken from each farm. The sum of the adjusted proportions multiplied by 100 gave the overall individual adjusted prevalence.

### Questionnaire design and administration

A pre-tested (pre-testing was done using seven pig farmers from two locations not included in the study areas) structured, interviewer-administered questionnaire was used to obtain data on demography, environmental and management factors, bio-security measures and ASF related signs. The questionnaires, containing 38 questions, were administered on farms where samples were obtained. A respondent was someone who was actively involved in the daily activities of the farm and was not necessarily the farm owner.

### Statistical analysis

We conducted descriptive statistics and univariate analysis using SAS version 9.3 [43]. We determined the odds ratio and statistical significance between seropositive and seronegative pig farms using Fisher’s exact test for discrete variables at the 95 % confidence level. Multivariable unconditional logistic regression was used to determine predictors for ASF seropositivity controlling for other covariates at *P* < 0.20 and biologically plausible ones such as feeding of swill, lending out boars for breeding and herd size. We tested for collinearity among predictors using the Chi square test for binomial variables. We also tested for interactions between selected variables. We used Akaike’s information criterion in selecting the variables. A forward selection method was used. The goodness of fit of the model was tested using the Pearson goodness of fit test. In the final models, only variables or interactions that were found to significantly affect the outcome at *P* < 0.05, and corresponding lower-order interactions terms whether significant or not, were retained. We determined the overall average compliance with standard biosecurity measures by calculating the average compliance level of the 15 internal biosecurity measures (bio-management) considered.

### Ethical considerations

This study was carried out in compliance with the guidelines of the Animal Ethics Committee of the Faculty of Veterinary Medicine, University of Ibadan, Nigeria. Informed consent was obtained from all participating pig farm owners.
